# Supply and Geographic Distribution of Geriatric Physicians and Geriatric Nurse Practitioners

**DOI:** 10.1001/jamanetworkopen.2024.44659

**Published:** 2024-11-13

**Authors:** Ying Xue, Lusine Poghosyan, Qinyun Lin

**Affiliations:** 1 School of Nursing, University of Rochester, Rochester, New York; 2 School of Nursing, Columbia University, New York, New York; 3 School of Public Health and Community Medicine, Institute of Medicine, University of Gothenburg, Gothenburg, Sweden

## Abstract

**Importance:**

The rapidly growing population of older adults and their concomitant high prevalence of chronic health conditions require an increased supply in the specialized geriatric workforce to meet increasing health care demands. Understanding trends and geographic disparities in the supply of the geriatric workforce is essential for developing effective policies.

**Objectives:**

To examine temporal and geographic trends in the supply of geriatric physicians (GMDs) and geriatric nurse practitioners (GNPs) from 2010 to 2020 and to assess potential disparities between metropolitan and nonmetropolitan counties.

**Design, Setting, and Participants:**

This repeated cross-sectional study used annual county-level data from 2010 to 2020, encompassing all counties in the 50 US states and Washington, DC. Statistical analysis was performed from June 2023 to March 2024.

**Main Outcomes and Measures:**

The primary outcomes were the numbers of GMDs, GNPs, and the combined number of GMDs and GNPs per 100 000 older adults. The secondary outcome included the proportion of counties with or without any GMDs or GNPs.

**Results:**

From 2010 to 2020, the national per capita supply of GMDs decreased by 12.7%, from 13.4 per 100 000 older adults in 2010 to 11.7 per 100 000 older adults in 2020, while GNPs increased by 125.0%, from 4.4 per 100 000 older adults in 2010 to 9.9 per 100 000 older adults in 2020. The combined GMD and GNP workforce increased by 21.3%, from 17.8 per 100 000 older adults in 2010 to 21.6 per 100 000 older adults in 2020. The distributions of older adults, GMDs, and GNPs closely resembled the distribution of metropolitan and nonmetropolitan counties, with GMDs and GNPs highly concentrated in metropolitan counties where the number of older adults was greatest. Throughout the study period, 63.9% of counties (2008 of 3142 in 2010-2019; 2009 of 3143 in 2020), predominantly small and nonmetropolitan counties, had no GMDs or GNPs, which was associated with the disparities between metropolitan and nonmetropolitan counties.

**Conclusions and Relevance:**

This repeated cross-sectional study found that from 2010 to 2020, the overall national supply of GMDs and GNPs kept pace with the growth of the older population, largely due to the rapid growth in the number of GNPs. However, significant geographic disparities persisted, particularly in small and nonmetropolitan counties. Future efforts should focus on increasing the availability of GMDs and GNPs in underserved small and nonmetropolitan counties to ensure equitable access to geriatric care.

## Key Points

**Questions:**

What are the temporal and geographic trends in the supply of geriatric physicians (GMDs) and geriatric nurse practitioners (GNPs), and are there disparities between metropolitan and nonmetropolitan counties?

**Findings:**

In this repeated cross-sectional study from 2010 to 2020, the national per capita supply of GMDs decreased by 12.7%, while GNPs increased by 125.0%; the combined GMD and GNP supply increased by 21.3%. Throughout the study period, 63.9% of counties, primarily small and nonmetropolitan counties, had no GMDs or GNPs.

**Meaning:**

This study suggests that the combined GMD and GNP workforce kept pace with the growth of the older population; however, considerable disparities existed between metropolitan and nonmetropolitan counties.

## Teaser

This repeated cross-sectional study examines temporal and geographic trends in the supply of geriatric physicians and geriatric nurse practitioners from 2010 to 2020 and assesses potential disparities between metropolitan and nonmetropolitan counties.

## Introduction

The US experienced the fastest growth of its older population from 2010 to 2020 since the decade spanning 1880 to 1890, and this trend is projected to continue.^1,2^ In 2020, 16.8% of the US population was 65 years of age or older (55.8 million), and by 2040, it is estimated it will increase to 21.6% (80.8 million).^1,2^ As more people live longer, they are disproportionally affected by chronic health conditions.^3,4^ The growing number of older adults and the concomitant high prevalence of chronic conditions require an increased supply of specialized geriatric clinicians with advanced training in aging and health.

The US Department of Health and Human Services estimated that “demand for geriatricians is projected to exceed supply, resulting in a national shortage of 26 980 FTEs [full-time equivalents] in 2025.”^5^ Geriatricians are geriatric physicians (GMDs) who specialize in providing medical care to older adults.^6^ Professional organizations such as the National Academy of Medicine and the American Geriatrics Society have called for addressing shortages of GMDs and ensuring that all geriatric professionals, including physicians, physician assistants (PAs), nurses, and social workers, are prepared to meet the unique and increasing health care needs of older adults.^7,8^ Geriatric nurse practitioners (GNPs) are 1 type of specialty nurse practitioners (NPs) who are certified in gerontology.^9,10^ In recent decades, the numbers of NPs, including GNPs, have increased significantly.^11,12^

Presently, little is known about the trends in the combined GMD and GNP (GMDNP) workforce, particularly the geographic distribution of GMDs and GNPs in association with older adults. In response to the rapidly growing population of older adults, it is necessary to understand the corresponding trends and geographic distribution in the geriatric workforce so that effective strategies and workforce development policies can be designed to meet the increasing demand for care by the aging population. The aim of this study was to examine temporal and geographic trends in the supply of GMDs and GNPs from 2010 to 2020 and to assess potential disparities between metropolitan and nonmetropolitan counties.

## Methods

We designed a repeated cross-sectional study covering the period from 2010 to 2020. An annual county-level geospatial dataset was constructed for the 50 US states and Washington, DC, using data from 2 national datasets: the Area Health Resources File (AHRF) and the National Provider Identifier (NPI) registry. The AHRF is a comprehensive dataset that includes county-level population, economic, health professional, and health facility data.^13^ The NPI registry contains information on all health care professionals who have had financial transactions with the Centers for Medicare & Medicaid Services, including their state-level practice certification.^14^ Because these datasets are publicly available with deidentified information, this study was granted exempt status by the University of Rochester institutional review board and was conducted in accordance with the Strengthening the Reporting of Observational Studies in Epidemiology (STROBE) reporting guideline.^15^

### Study Variables

Data on county characteristics were extracted from the AHRF, whereas data on GMDs and GNPs were extracted from the NPI registry. Both GMDs and GNPs were identified through self-claimed clinical specialties. Specialties for GMDs included geriatric family medicine, geriatric internal medicine, and geriatric psychiatry^6^; the specialty for GNPs was gerontology.^10^ The study included health care professionals who were registered as individuals in the NPI registry and whose registration remained active for 12 months in a given study year.

We defined older adults as those aged 65 years or older. The primary outcomes were the densities of GMDs, GNPs, and GMDNPs, calculated separately as the number of each per 100 000 older adults. The secondary outcomes included the proportion of counties with or without any GMDs or GNPs. We examined various county characteristics, including population, socioeconomics, health resources, and geography. County metropolitan status was defined as those located in metropolitan areas based on the 2023 Rural-Urban Continuum Codes (codes 1-3).^16^

### Statistical Analysis

Statistical analysis was performed from June 2023 to March 2024. Descriptive statistics were calculated for the numbers of GMDs, GNPs, GMDNPs, and older adults, as well as the densities of GMDs, GNPs, and GMDNPs at the national and county levels. To examine the temporal trends in GMD, GNP, and GMDNP densities overall and across metropolitan and nonmetropolitan areas in counties with any GMDs or GNPs from 2010 to 2020, we used a growth curve modeling approach suitable for analyzing repeated cross-sectional data. For each trend analysis, a 3-level random intercept multilevel model (time points, counties, and states) was constructed, adjusting for state-level clustering effect. This model also assessed whether the temporal trends were different between metropolitan and nonmetropolitan counties and compared the temporal trends between GMD density and GNP density. Descriptive statistics were also computed for county characteristics in 2020 between counties with and counties without GMDs or GNPs from 2010 to 2020. To assess whether each county characteristic differed between these 2 groups, we conducted bivariate analysis for each characteristic using a 2-level random intercept multilevel model (counties and states). All statistical tests were 2-sided, and results were deemed statistically significant at *P* < .05. Analyses were performed using SAS, version 9.4 (SAS Institute Inc).

## Results

There were 3142 US counties before 2020 and 3143 US counties in 2020 as 1 county was split into 2 in 2020. County metropolitan status did not change over time. Of these, 1166 counties were metropolitan and 1976 counties (1977 in 2020) were nonmetropolitan.

**Figure 1.**
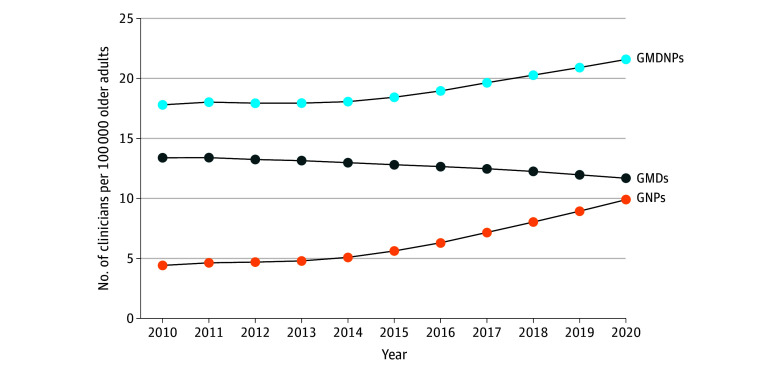
Trends in National-Level Densities of Geriatric Physicians (GMDs), Geriatric Nurse Practitioners (GNPs), and Combined GMDs and GNPs (GMDNPs) in the US, 2010-2020 The density is defined as the number of GMDs, GNPs, or GMDNPs per 100 000 older adults.

Nationally, the number of GMDs increased by 20.7% from 5388 in 2010 to 6501 in 2020, while the number of GNPs increased by 209.8% from 1778 to 5508; concurrently, the number of older adults increased by 38.2% from 40 266 647 to 55 659 365. Figure 1 illustrates that GMD density decreased by 12.7%, from 13.4 per 100 000 older adults in 2010 to 11.7 per 100 000 older adults in 2020. Conversely, GNP density increased by 125.0%, from 4.4 per 100 000 older adults in 2010 to 9.9 per 100 000 older adults in 2020. Collectively, GMDNP density increased by 21.3%, from 17.8 per 100 000 older adults in 2010 to 21.6 per 100 000 older adults in 2020.

**Figure 2.**
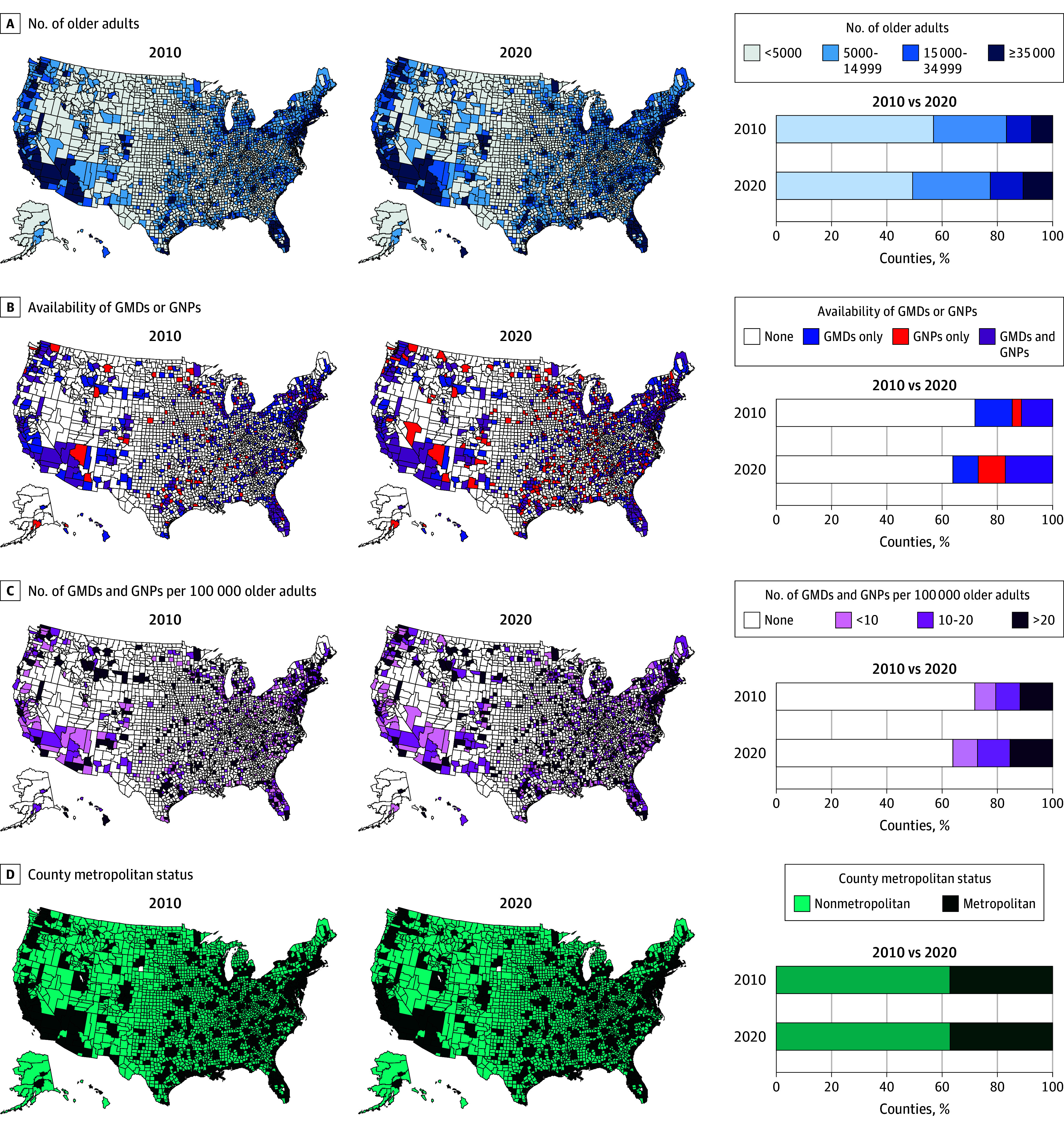
Geographic Trends in Older Adults, Geriatric Physicians (GMDs), and Geriatric Nurse Practitioners (GNPs) Among US Counties: 2010 vs 2020

Figure 2 displays the geographic distributions of older adults, GMDs, and GNPs for the years 2010 and 2020. Several distinct patterns emerged. First, the distributions of older adults, GMDs, and GNPs closely resembled the distribution of metropolitan and nonmetropolitan counties, with GMDs and GNPs highly concentrated in metropolitan counties where the number of older adults was greatest. Second, a large proportion of counties (71.9% in 2010; 63.9% in 2020) did not have any GMDs or GNPs; these counties were mostly nonmetropolitan (75.9% in 2010; 78.4% in 2020). Third, compared with the distribution in 2010, there were notable changes in 2020: the proportion of counties with fewer than 5000 older adults decreased (from 56.9% [1788] to 49.3% [1550]); the proportion of counties with 35 000 older adults or more increased (from 7.6% [240] to 10.7% [335]); the proportion of counties without any GMDs or GNPs decreased (from 71.9% [2258] to 63.9% [2009]); the proportion of counties with both GMDs and GNPs increased (from 11.3% [355] to 17.1% [538]); and the proportion of counties with GMDNP density greater than 20 per 100 000 older adults increased (from 11.7% [368] to 15.4% [483]).

**Figure 3.**
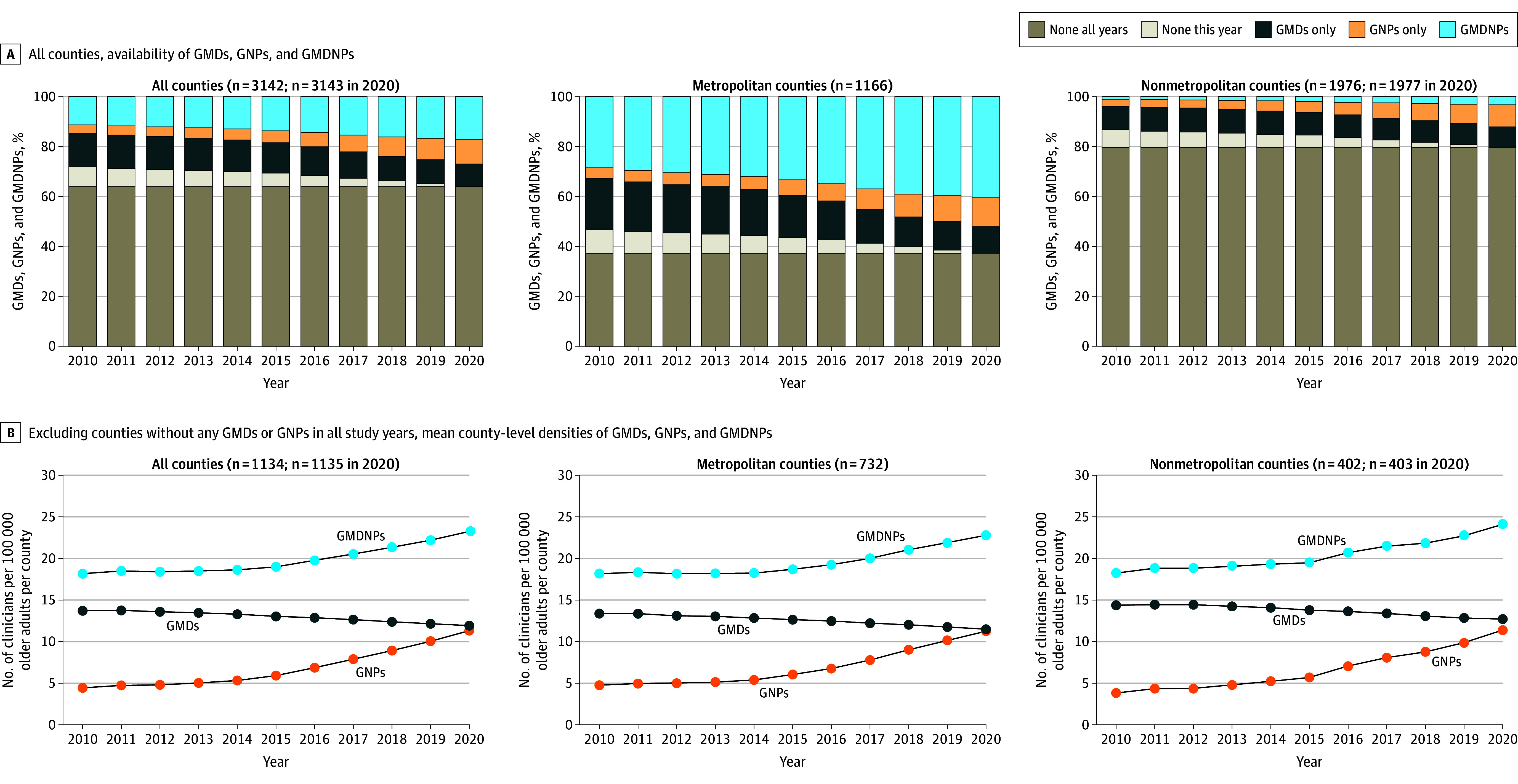
Trends in County-Level Geriatric Physicians (GMDs), Geriatric Nurse Practitioners (GNPs), and Combined GMDs and GNPs (GMDNPs) by County Metropolitan Status in the US, 2010-2020 The density is defined as the number of GMDs, GNPs, or GMDNPs per 100 000 older adults.

Figure 3A shows the availability of GMDs or GNPs by county metropolitan status from 2010 to 2020. To better understand the changes over time, we counted separately the counties without any GMDs or GNPs in all study years vs counties without any GMDs or GNPs in only a single study year. The proportion of counties without any GMDs or GNPs in all study years was 63.9% (2008 in 2010-2019; 2009 in 2020); this proportion differed by metropolitan status: 37.2% (434) in metropolitan counties and 79.7% (1574 in 2010-2019; 1575 in 2020) in nonmetropolitan counties. From 2010 to 2020, the proportion of counties without any GMDs or GNPs in only a single study year decreased from 8.0% (250) to 0 for all counties, 9.4% (110) to 0 in metropolitan counties, and 7.1% (140) to 0 in nonmetropolitan counties. The proportion of counties with only GMDs decreased from 13.5% (425) to 9.2% (288) in all counties, 20.6% (240) to 10.7% (125) in metropolitan counties, and 9.4% (185) to 8.2% (163) in nonmetropolitan counties. The proportion of counties with only GNPs increased from 3.3% (104) to 9.8% (308) in all counties, 4.2% (49) to 11.5% (134) in metropolitan counties, and 2.8% (55) to 8.8% (174) in nonmetropolitan counties. The proportion of counties with both GMDs and GNPs increased from 11.3% (355) to 17.1% (538) in all counties, 28.6% (333) to 40.6% (473) in metropolitan counties, and 1.1% (22) to 3.3% (65) in nonmetropolitan counties.

Figure 3B shows the trends in densities of GMDs, GNPs, and GMDNPs by county metropolitan status from 2010 to 2020, after excluding counties without any GMDs or GNPs in all study years. The trends were similar between metropolitan and nonmetropolitan counties; GMD density decreased, GNP density increased, and GMDNP density increased. Regardless of county metropolitan status, GNP density outpaced that of GMD density (eTables 1-3 in Supplement 1).

Table 1 presents the mean and median densities per 100 000 older adults of GMDs, GNPs, and GMDNPs, along with the annual growth rate (AGR) in mean density among counties with GMDs or GNPs during the study period. The GMD mean density decreased from 13.7 (95% CI, 12.6-14.9) in 2010 to 11.9 (95% CI, 10.9-12.9) in 2020 when pooling both metropolitan and nonmetropolitan counties (AGR, −1.5%; 95% CI, −1.6% to −1.3%). In metropolitan counties, the GMD mean density decreased from 13.4 (95% CI, 12.2-14.6) in 2010 to 11.5 (95% CI, 10.5-12.5) in 2020 (AGR, −1.5%; 95% CI, −1.7% to −1.3%), and in nonmetropolitan counties, it decreased from 14.4 (95% CI, 11.9-16.8) in 2010 to 12.7 (95% CI, 10.6-14.9) in 2020 (AGR, −1.4%; 95% CI, −1.6% to −1.2%). The GNP mean density increased from 4.4 (95% CI, 3.9-5.0) in 2010 to 11.3 (95% CI, 10.5-12.1) in 2020 in the combined metropolitan and nonmetropolitan counties (AGR, 9.8%; 95% CI, 9.3%-10.2%). In metropolitan counties, the GNP mean density increased from 4.8 (95% CI, 4.2-5.4) in 2010 to 11.3 (95% CI, 10.3-12.2) in 2020 (AGR, 9.2%; 95% CI, 8.7%-9.7%), and in nonmetropolitan counties, it increased from 3.8 (95% CI, 2.9-4.8) in 2010 to 11.4 (95% CI, 10.0-12.8) in 2020 (AGR, 11.0%; 95% CI, 10.0%-12.1%). The GMDNP mean density increased from 18.2 (95% CI, 16.9-19.5) in 2010 to 23.2 (95% CI, 21.9-24.5) in 2020 in the combined metropolitan and nonmetropolitan counties (AGR, 2.5%; 95% CI, 2.3%-2.6%). In metropolitan counties, the GMDNP mean density increased from 18.2 (95% CI, 16.6-19.7) in 2010 to 22.8 (95% CI, 21.1-24.4) in 2020 (AGR, 2.3%; 95% CI, 2.2%-2.5%), and in nonmetropolitan counties, it increased from 18.2 (95% CI, 15.7-20.7) in 2010 to 24.1 (95% CI, 21.9-26.3) in 2020 (AGR, 2.7%; 95% CI, 2.3%-3.0%). Comparing the annual growth between metropolitan and nonmetropolitan counties, only the growth in GNP density was statistically significantly different, with the increase being lower in metropolitan counties than in nonmetropolitan counties (−2.0%; 95% CI, −2.9% to −1.0%).

**Table 1.** County-Level Mean and Median Values of GMD, GNP, and GMDNP Densities by County Metropolitan Status Among Counties With Any GMDs or GNPs During the Study Period in the US, 2010-2020

Characteristic	2010		2020		From 2010 to 2020^a^			
	Mean density (95% CI)	Median density (IQR)	Mean density (95% CI)	Median density (IQR)	Annual growth rate in mean density, % (95% CI)	*P* value for trend	Year × (metropolitan vs nonmetropolitan), % (95% CI)	*P* value for trend
**GMD density**									
Metropolitan and nonmetropolitan	13.7 (12.6 to 14.9)	8.4 (0.0 to 18.3)	11.9 (10.9 to 12.9)	7.3 (0.0 to 15.8)	−1.5 (−1.6 to −1.3)	<.001	−0.1 (−0.4 to 0.2)	.40
Metropolitan	13.4 (12.2 to 14.6)	9.0 (3.7 to 18.1)	11.5 (10.5 to 12.5)	7.6 (3.2 to 14.9)	−1.5 (−1.7 to −1.3)	<.001		
Nonmetropolitan	14.4 (11.9 to 16.8)	6.3 (0.0 to 20.1)	12.7 (10.6 to 14.9)	6.9 (0.0 to 17.0)	−1.4 (−1.6 to −1.2)	<.001		
**GNP density**									
Metropolitan and nonmetropolitan	4.4 (3.9 to 5.0)	0.0 (0.0 to 5.8)	11.3 (10.5 to 12.1)	8.2 (0.0 to 15.7)	9.8 (9.3 to 10.2)	<.001	−2.0 (−2.9 to −1.0)	<.001
Metropolitan	4.8 (4.2 to 5.4)	1.6 (0.0 to 6.6)	11.3 (10.3 to 12.2)	8.3 (3.8 to 14.9)	9.2 (8.7 to 9.7)	<.001		
Nonmetropolitan	3.8 (2.9 to 4.8)	0.0 (0.0 to 0.0)	11.4 (10.0 to 12.8)	7.9 (0.0 to 18.2)	11.0 (10.0 to 12.1)	<.001		
**GMDNP density**									
Metropolitan and nonmetropolitan	18.2 (16.9 to 19.5)	12.8 (4.5 to 24.2)	23.2 (21.9 to 24.5)	17.2 (10.0 to 27.8)	2.5 (2.3 to 2.6)	<.001	−0.3 (−0.7 to 0.02)	.06
Metropolitan	18.2 (16.6 to 19.7)	12.7 (5.9 to 23.4)	22.8 (21.1 to 24.4)	16.3 (9.1 to 27.7)	2.3 (2.2 to 2.5)	<.001		
Nonmetropolitan	18.2 (15.7 to 20.7)	13.2 (0.0 to 25.2)	24.1 (21.9 to 26.3)	19.1 (11.9 to 27.8)	2.7 (2.3 to 3.0)	<.001		

Abbreviations: GMD, geriatric physician; GMDNP, combined GMD and GNP; GNP, geriatric nurse practitioner.

^a^ The results were estimated from the 3-level random intercept multilevel models. In 2010, metropolitan, n = 732; nonmetropolitan, n = 402. In 2020, metropolitan, n = 732; nonmetropolitan, n = 403. The density is defined as the number of GMDs, GNPs, or GMDNPs per 100 000 older adults.

Table 2 presents county characteristics in 2020 for counties without any GMDs or GNPs in all study years compared with those with GMDs or GNPs. In comparison with counties with GMDs or GNPs, counties without GMDs or GNPs had significantly fewer older adults and slightly higher percentages of older adults and non-Hispanic White residents. Socioeconomically, these counties had a higher percentage of residents without a high school diploma, a lower unemployment rate, and a lower median household income. Regarding health resources, counties without GMDs or GNPs had fewer physicians, NPs, and PAs from all specialties per 100 000 population. Except for rural health clinics (RHCs), these counties had a significantly lower proportion with hospitals, licensed nursing homes, skilled nursing facilities, home health agencies, community health centers (CHCs), and National Health Services Corps (NHSC) sites with health care professionals. Geographically, counties without GMDs or GNPs were predominately nonmetropolitan, while counties with GMDs or GNPs were mostly metropolitan.

**Table 2.** US County Characteristics in 2020 by Availability of GMDs or GNPs During the Study Period of 2010-2020

County characteristic	Counties without GMDs or GNPs during 2010-2020 (n = 2009)	Counties with GMDs or GNPs during 2010-2020 (n = 1134)	*P* value^a^
Population, mean (95% CI)			
Total county population, No.	22 799 (21 626-23 972)	250 159 (219 679-280 640)	<.001
Older adults, No.	4423 (4229-4618)	41 246 (36 671-45 820)	<.001
Older adults, %	21.1 (20.9-21.3)	18.6 (18.3-18.9)	<.001
Hispanic population, %	9.3 (8.6-9.9)	11.1 (10.3-11.9)	<.001
Non-Hispanic Black population, %	8.0 (7.3-8.6)	10.9 (10.1-11.7)	<.001
Non-Hispanic White population, %	77.5 (76.6-78.4)	72.0 (70.9-73.2)	<.001
Socioeconomic characteristics, mean (95% CI)			
Residents without high school diploma, %	13.2 (12.9-13.5)	11.0 (10.7-11.2)	<.001
Unemployed, %	6.4 (6.3-6.5)	7.3 (7.2-7.4)	.03
Median household income, $	53 994 (53 486-54 501)	63 613 (62 617-64 609)	<.001
Health resources			
Total No. of physicians from all specialties per 100 000 population, mean (95% CI)	83.1 (79.8-86.5)	248.8 (233.3-264.3)	<.001
Total No. of NPs from all specialties per 100 000 population, mean (95% CI)	63.1 (61.1-65.2)	94.9 (91.5-98.4)	<.001
Total No. of PAs from all specialties per 100 000 population, mean (95% CI)	26.4 (24.9-27.9)	42.9 (40.0-45.9)	<.001
Counties with hospitals, No. (%)	1403 (69.8)	1063 (93.7)	<.001
Counties with licensed nursing homes, No. (%)	177 (8.8)	204 (18.0)	<.001
Counties with skilled nursing facilities, No. (%)	1707 (85.0)	1121 (98.9)	<.001
Counties with home health agencies, No. (%)	876 (43.6)	943 (83.2)	<.001
Counties with rural health clinics, No. (%)	1319 (65.7)	488 (43.0)	<.001
Counties with community health centers, No. (%)	1129 (56.2)	938 (82.7)	<.001
Counties with NHSC site with health care professionals, No. (%)	885 (44.1)	791 (69.8)	<.001
Geography, No. (%)			
Metropolitan status			
Metropolitan	434 (21.6)	732 (64.6)	<.001
Nonmetropolitan	1575 (78.4)	402 (35.4)	
Region			
Midwest	742 (36.9)	313 (27.6)	<.001
Northeast	56 (2.8)	161 (14.2)	
South	921 (45.8)	501 (44.2)	
West	290 (14.4)	159 (14.0)	

Abbreviations: GMDs, geriatric physicians; GNPs, geriatric nurse practitioners; NHSC, National Health Services Corps; NPs, nurse practitioners; PAs, physician assistants.

^a^ Results were from the bivariate analysis for each characteristic using a 2-level random intercept multilevel model.

## Discussion

Our findings have several key implications for geriatric workforce policy. First, on the national level, the overall supply of GMDNPs has kept pace with the growth of the older population. Our data show a trend in increasing GMDNP density despite a trend in decreasing GMD density. The decreasing GMD density is consistent with existing evidence,^5^ which has sparked concerns about having insufficient GMDs to care for the growing population of older adults.^17^ However, the trend in increasing GMDNP density indicates that the overall growth in the combined number of GMDs and GNPs has exceeded the growth of the older population. This finding is encouraging in addressing the health care needs of older adults. The increase in GMDNP density is largely associated with the exponential growth in GNP density, indicating that GNPs play a critical role in expanding the geriatric workforce and maintaining the required supply of geriatric clinicians. The higher growth in NPs compared with physicians is not unique to geriatrics and has been observed in primary care and health care overall.^18,19^ Previous evidence has shown that care provided by GNPs was associated with reduced risk of hospital readmission and lower rates of depression, urinary incontinence, pressure ulcers, restraint use, and aggressive behaviors, as well as increased patient and caregiver satisfaction in long-term care settings.^20,21,22^ Our study findings support the call by professional organizations to ensure all geriatric professionals are adequately prepared to meet the increasing health care needs of the older population.^7,8^

Second, while the national supply of GMDNPs has kept pace with the growth in the older adult population, our study revealed marked geographic disparities in the availability of GMDs and GNPs across metropolitan and nonmetropolitan counties. Geographic maldistribution of the health workforce is not a new issue.^23,24^ However, the extent of counties without any GMDs or GNPs over the 11-year study period was extensive. We found that 63.9% of counties had no GMDs or GNPs in any of the study years where 8.9 million older adults resided in 2020. Our data show that these counties were primarily nonmetropolitan with small populations of older adults (fewer than 5000), lower socioeconomic status, and significantly lower densities of physicians, NPs, and PAs from all specialties, as well as a lower proportion with health care facilities. Furthermore, most of these counties were geographically clustered (Figure 2B), creating additional challenges for accessing geriatric care.

The absence of any GMDs or GNPs in many small and nonmetropolitan counties is the primary factor associated with the geriatric workforce disparities between metropolitan and nonmetropolitan counties. After excluding counties without any GMDs or GNPs in all study years, the trends in GMD and GMDNP densities were parallel between metropolitan and nonmetropolitan counties, with a trend toward a higher GNP density in nonmetropolitan compared with metropolitan counties.

Future efforts should focus on increasing the availability of GMDs or GNPs in small and nonmetropolitan counties to improve equity in access to geriatric care. It is evident that the size of the population of older adults and its health conditions determine the demand for geriatric care, which drives the business of geriatric care and the employment of geriatric clinicians. In counties with fewer than 5000 older adults and lower socioeconomic status, it is challenging for health care facilities to maintain financial viability. The increasing rate of rural hospital closures and the higher number of nursing home closures in nonmetropolitan areas further exacerbate this issue.^25,26^ Our data show that these counties had a significantly lower proportion with health care facilities, with the exception of RHCs. Federal programs have been established to improve the health infrastructure in rural and medically underserved areas, such as RHCs and CHCs, and incentivize clinicians to practice in these areas through scholarships and student loan repayments in exchange for service in NHSC sites.^27^ Our data demonstrate the effectiveness of these efforts in supporting RHCs. However, a significantly lower proportion of these counties had CHCs and NHSC sites. Future research is needed to identify more effective solutions to address these disparities.

Third, our study findings show slight increases in the number of counties with GNPs only and those with both GMDs and GNPs. The number of counties with both GMDs and GNPs increased from 355 to 538 between 2010 and 2020. Although modest, this upward trend aligns with the growing practice of involving both physicians and NPs in primary care and long-term care settings.^28,29^ Evidence indicates that optimal staffing models to care for frail older adults, with regard to service provision and costs in primary care and geriatrics practices, involve a higher proportion of NPs relative to physicians.^30^

Fourth, among the counties with GMDs or GNPs, the GNP density grew faster in nonmetropolitan counties than in metropolitan counties. This finding is promising given the need to increase the availability of GMDs or GNPs in small and nonmetropolitan counties. Considering the small number of counties with only GNPs, continued monitoring of this trend is warranted.

### Limitations

The study has several limitations. First, we assessed only GMDs and GNPs, which are part of the overall geriatric workforce, as we did not have data on other geriatric clinicians, such as geriatric PAs. The interpretation of our findings should be held within this context. Nonetheless, because GMDs and GNPs comprise the largest part of the geriatric workforce, our study findings hold constructive implications. Second, using clinical certification to identify GMDs or GNPs might not accurately classify all physicians or NPs practicing in geriatrics. It is possible that some physicians or NPs practice in geriatrics but do not hold certification in geriatrics or gerontology.^31,32,33^ Third, data for GMDs and GNPs were extracted from the self-reported NPI database, which does not mandate updates and might contain obsolete information.^34^ These limitations could lead to a slight misestimation of the number of actively practicing GMDs or GNPs and subsequent modest misclassifications in their distribution. However, these limitations are minor and are unlikely to have a significant association with the validity of study results. We compared the trend of GMDs identified in the NPI data with the trend of certified GMDs reported by the American Board of Medical Specialties^35^ and found consistent results. We could not assess the validity of GNP data because there are no other national-level GNP data available for comparison. However, the trend in the increasing number of GNPs found in this study is consistent with the trend in the increasing number of NPs in primary care.^19^

## Conclusions

In this repeated cross-sectional study of GMDs and GNPs, the overall combined national supply of GMDs and GNPs kept pace with the growth of the older population from 2010 to 2020 owing to the fast growth in GNPs. However, considerable geographic disparities existed in the availability of GMDs and GNPs between metropolitan and nonmetropolitan counties. These disparities were primarily due to the disproportionately high number of small and nonmetropolitan counties that did not have any GMDs or GNPs in all study years. Future efforts should focus on increasing the numbers of GMDs and GNPs in those counties to improve equity in access to geriatric care.

## Notes

Supplement 1. **eTable 1.** Trend in GNP Density Versus GMD Density Among Counties With Any GMDs or GNPs During the Study Period in the US, 2010-2020 **eTable 2.** Trend in GNP Density Versus GMD Density Among Metropolitan Counties With Any GMDs or GNPs During the Study Period in the US, 2010-2020 **eTable 3.** Trend in GNP Density Versus GMD Density Among Nonmetropolitan Counties With Any GMDs or GNPs During the Study Period in the US, 2010-2020

Supplement 2.
Data Sharing Statement
