# Supplementary material for: Supply and Geographic Distribution of Geriatric Physicians and Geriatric Nurse Practitioners
Source: JAMA Netw Open. 2024 Nov 13;7(11):e2444659. doi: 10.1001/jamanetworkopen.2024.44659 (PMC11561691; doi:10.1001/jamanetworkopen.2024.44659)
Supplement: Supplement 1. — eTable 1. Trend in GNP Density Versus GMD Density Among Counties With Any GMDs or GNPs During the Study Period in the US, 2010-2020 eTable 2. Trend in GNP Density Versus GMD Density Among Metropolitan Counties With Any GMDs or GNPs During the Study Period in the US, 2010-2020 eTable 3. Trend in GNP Density Versus GMD Density Among Nonmetropolitan Counties With Any GMDs or GNPs During the Study Period in the US, 2010-2020 [file jamanetwopen-e2444659-s001.pdf]

Supplementary Online Content

Xue Y, Poghosyan L, Lin Q. Supply and geographic distribution of geriatric physicians and geriatric nurse practitioners. *JAMA Netw Open.* 2024;7(11):e2444659. doi:10.1001/jamanetworkopen.2024.44659

eTable 1. Trend in GNP Density Versus GMD Density Among Counties With Any GMDs or GNPs During the Study Period in the US, 2010-2020

eTable 2. Trend in GNP Density Versus GMD Density Among Metropolitan Counties With Any GMDs or GNPs During the Study Period in the US, 2010-2020

eTable 3. Trend in GNP Density Versus GMD Density Among Nonmetropolitan Counties With Any GMDs or GNPs During the Study Period in the US, 2010-2020

This supplementary material has been provided by the authors to give readers additional information about their work.

© 2024 Xue Y et al. *JAMA Network Open.*

eTable 1. Trend in GNP Density Versus GMD Density Among Counties With Any GMDs or GNPs During the Study Period in the US, 2010-2020

Variable	Estimate	95% CI		*P*
Intercept	2.6799	2.5732	2.7866	<.0001
GNP	-1.249	-1.2997	-1.1983	<.0001
GMD	ref			
Year	-0.0148	-0.0207	-0.0089	<.0001
GNP * Year	0.1121	0.1036	0.1207	<.0001
GMD * Year	ref			

Note. CI: confidence interval; GMD: geriatric physician; GNP: geriatric nurse practitioner. The density is defined as the number of GMDs, GNPs, or combined GMDs and GNPs per 100 000 older adults.

© 2024 Xue Y et al. *JAMA Network Open.*

eTable 2. Trend in GNP Density Versus GMD Density Among Metropolitan Counties With Any GMDs or GNPs During the Study Period in the US, 2010-2020

Variable	Estimate	95% CI		*P*
Intercept	2.6163	2.5113	2.7214	<.001
GNP	-1.1699	-1.2176	-1.1223	<.001
GMD	ref			
Year	-0.0153	-0.0208	-0.0099	<.001
GNP * Year	0.1066	0.0986	0.1145	<.001
GMD * Year	ref			

Note. CI: confidence interval; GMD: geriatric physician; GNP: geriatric nurse practitioner. The density is defined as the number of GMDs, GNPs, or combined GMDs and GNPs per 100 000 older adults.

© 2024 Xue Y et al. *JAMA Network Open.*

eTable 3. Trend in GNP Density Versus GMD Density Among Nonmetropolitan Counties With Any GMDs or GNPs During the Study Period in the US, 2010-2020

Variable	Estimate	95% CI		*P*
Intercept	2.7484	2.5507	2.9462	<.001
GNP	-1.4022	-1.5162	-1.2883	<.001
GMD	ref			
Year	-0.0140	-0.0274	-0.0006	0.04
GNP * Year	0.1242	0.105	0.1433	<.001
GMD * Year	ref			

Note. CI: confidence interval; GMD: geriatric physician; GNP: geriatric nurse practitioner. The density is defined as the number of GMDs, GNPs, or combined GMDs and GNPs per 100 000 older adults.

© 2024 Xue Y et al. *JAMA Network Open.*
