# Supplementary material for: Supply and Geographic Distribution of Geriatric Physicians and Geriatric Nurse Practitioners
Source: JAMA Netw Open. 2024 Nov 13;7(11):e2444659. doi: 10.1001/jamanetworkopen.2024.44659 (PMC11561691; doi:10.1001/jamanetworkopen.2024.44659)
Supplement: Supplement 2. — Data Sharing Statement [file jamanetwopen-e2444659-s002.pdf]

Data Sharing Statement

Xue. Supply and Geographic Distribution of Geriatric Physicians and Geriatric Nurse Practitioners. *JAMA Netw Open*. Published November 13, 2024. doi:10.1001/jamanetworkopen.2024.44659

Data
Data available: Yes
Data types: Deidentified participant data
How to access data: Area Health Resources File were published by the U.S. Department of Health and Human Services and can be accessed at https://data.hrsa.gov/topics/health-workforce/ahrf; National Provider Identifier registry was published by the Center for Medicare and Medicaid Services and can be accessed at https://nppes.cms.hhs.gov/#/
When available: With publication

Supporting Documents
Document types: None

Additional Information
Who can access the data: Data are publicly available
Types of analyses: Data are available for any type of analyses for any purpose
Mechanisms of data availability: Publicly available
